# In-cell identification and measurement of RNA-protein interactions

**DOI:** 10.1038/s41467-019-13235-w

**Published:** 2019-11-22

**Authors:** Antoine Graindorge, Inês Pinheiro, Anna Nawrocka, Allison C. Mallory, Peter Tsvetkov, Noa Gil, Carlo Carolis, Frank Buchholz, Igor Ulitsky, Edith Heard, Mikko Taipale, Alena Shkumatava

**Affiliations:** 1 Institut Curie, PSL Research University, CNRS UMR3215, INSERM U934, Paris, 75005 France; 2 Whitehead Institute for Biomedical Research, Cambridge, MA 02142 USA; 3 Department of Biological Regulation, Weizmann Institute of Science, Rehovot, 76100 Israel; 4 Biomolecular Screening and Protein Technologies Unit, Centre de Regulació Genòmica (CRG), The Barcelona Institute of Science and Technology, Barcelona, Spain; 5 Medical System Biology, UCC, Medical Faculty Carl Gustav Carus, TU Dresden, Dresden, Germany; 6 Max Planck Institute for Molecular Cell Biology and Genetics, Dresden, Germany; 7 Donnelly Centre for Cellular and Biomolecular Research, Department of Molecular Genetics, Toronto, Canada

**Keywords:** RNA, Non-coding RNAs

## Abstract

Regulatory RNAs exert their cellular functions through RNA-binding proteins (RBPs). Identifying RNA-protein interactions is therefore key for a molecular understanding of regulatory RNAs. To date, RNA-bound proteins have been identified primarily through RNA purification followed by mass spectrometry. Here, we develop incPRINT (in cell protein-RNA interaction), a high-throughput method to identify in-cell RNA-protein interactions revealed by quantifiable luminescence. Applying incPRINT to long noncoding RNAs (lncRNAs), we identify RBPs specifically interacting with the lncRNA *Firre* and three functionally distinct regions of the lncRNA *Xist*. incPRINT confirms previously known lncRNA-protein interactions and identifies additional interactions that had evaded detection with other approaches. Importantly, the majority of the incPRINT-defined interactions are specific to individual functional regions of the large *Xist* transcript. Thus, we present an RNA-centric method that enables reliable identification of RNA-region-specific RBPs and is applicable to any RNA of interest.

## Abstract (web summary)

RNA-interacting proteome can be identified by RNA affinity purification followed by mass spectrometry. Here the authors developed a different RNA-centric technology that combines high-throughput immunoprecipitation of RNA binding proteins and luciferase-based detection of their interaction with the RNA.

## Introduction

The identification of RNA–protein interactions is essential for deciphering the cellular functions and molecular mechanisms of regulatory RNAs that act through the formation of dynamic ribonucleoprotein (RNP) complexes^1^. There are several established protein-centric approaches such as cross-linking immunoprecipitation (CLIP) methods that reliably and systematically identify all transcripts bound by individual proteins of interest^2^. By contrast, when the aim is to determine the proteomes bound by individual transcripts, RNA-centric approaches are applied, which are mainly based on affinity capture of an RNA of interest followed by mass-spectrometry (MS) identification of the co-purified proteins^3^. These powerful methods have led to the identification of a number of functionally relevant RNA–protein interactions. Nevertheless, the unbiased identification of RNA-bound proteomes following probe-based RNA immunoprecipitation often remains challenging due to the generally low efficiency of RNA affinity purifications requiring scaling up of the starting cellular material. When applied to RNAs expressed at the low copy numbers characteristic of many lncRNAs^4^, generating sufficient input material for RNA affinity capture-MS is difficult and presents a risk of contamination by nonspecific RBPs. In addition, RBPs that are expressed at relatively low levels often do not reach the threshold required for their robust detection by mass spectrometry. Furthermore, nearly all currently available approaches enable the identification of proteins binding the full-length transcript of interest, whereas many lncRNAs have a modular organization with discrete RNA regions performing different functions^1,5^. While dChIRP based on RNA affinity capture-MS has allowed validation of already known RNA-region-specific protein interactions^6^, the de novo identification and assignment of RBPs bound to distinct RNA regions requires dedicated approaches including comparative RNA affinity capture-MS performed on a mutant variant of the RNA of interest^7^ or precise mapping of the individual RBP binding by protein-centric CLIP methods^8,9^.

Given the growing repertoire of RNAs with important biological functions and the experimental challenges inherent in dissecting their molecular mechanisms of action, complementary strategies could prove helpful for defining the protein interactions of individual RNAs. Here, we present incPRINT, an alternative RNA-centric method to facilitate the in-cell identification and measurement of RNA–protein interactions. One of the distinguishing features of incPRINT is that it does not utilize direct RNA affinity capture, but is rather based on high-throughput immunoprecipitation of thousands of RBPs followed by luciferase-based detection of their interactions with the RNA of interest. Applying incPRINT to well-studied lncRNAs as a proof-of-principle demonstrates that incPRINT enables the identification of proteins interacting with RNAs expressed at low endogenous levels as well as proteins interacting with distinct regions of RNA, facilitating the assignment of RNA-interaction domains. We thus introduce incPRINT as a reliable method to identify in-cell RNA-interacting proteomes.

## Results

### The incPRINT method to identify RNA–protein complexes

**Fig. 1:**
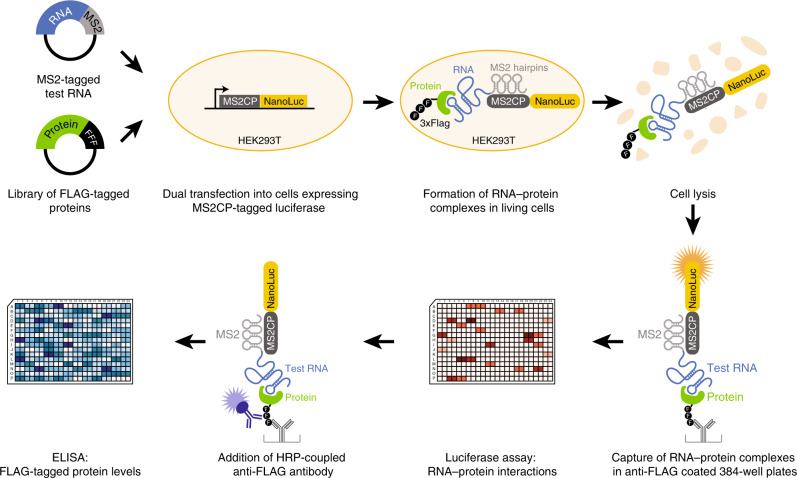
Principle of the incPRINT method. HEK293T cells, stably expressing a NanoLuc luciferase-MS2CP recombinant protein from an integrated plasmid, were co-transfected with plasmids encoding a MS2-tagged test RNA and 3xFLAG-tagged test proteins in a 96-well format (each well contains cells expressing one tagged test RNA and one tagged test protein). Cell lysates were applied to anti-FLAG-coated 384-well plates to immuno-purify test proteins with their interacting RNAs. After washing off nonspecific interactions, the MS2-RNA/FLAG-tagged protein complexes were detected by NanoLuc luciferase, tethered to the test RNA through the MS2-MS2CP interaction. The expression levels of the FLAG-tagged test proteins were detected by ELISA using a second anti-FLAG antibody coupled to horseradish peroxidase (HRP).

To identify RNA–protein interactions systematically in living cells, we developed a method that measures the cellular interactions between any tagged test RNA and any tagged test protein. The principle of incPRINT is the transient dual expression of a MS2-tagged test RNA and a FLAG-tagged test protein in HEK293T cells stably expressing a luciferase detector fused to the MS2 coat protein (MS2CP) from a genomically integrated plasmid (Fig. 1). The test RNA is tethered to the luciferase detector through the *MS2*-MS2CP interaction; the RNA-luciferase complex is co-purified with the FLAG-tagged test protein immunoprecipitated from cell lysates by anti-FLAG antibody (Fig. 1). Indirect RNA–protein interactions bridged by DNA are eliminated by DNase treatment after the cell lysis step. To detect each RNA–protein interaction, RNA-MS2 co-purified with the test FLAG-tagged protein is measured by quantifiable luciferase luminescence (Fig. 1). To control for test protein expression levels, the abundance of the test proteins is measured by ELISA using a second anti-FLAG antibody coupled to horseradish peroxidase (HRP) (Fig. 1). The incPRINT method is flexible in scale, usable as a low- or high-throughput assay. To enable systematic, high-throughput identification of cellular RNA–protein interactions, we generated a customized library of ∼3000 human FLAG-tagged proteins including ∼1500 known RBPs (based on refs. ^10,11^), ∼1300 transcription factors^12^, and ∼170 chromatin-associated proteins. The tagged protein content can be adapted to fit the desired experimental setup.

### incPRINT reliably detects cellular RNA–protein interactions

**Fig. 2:**
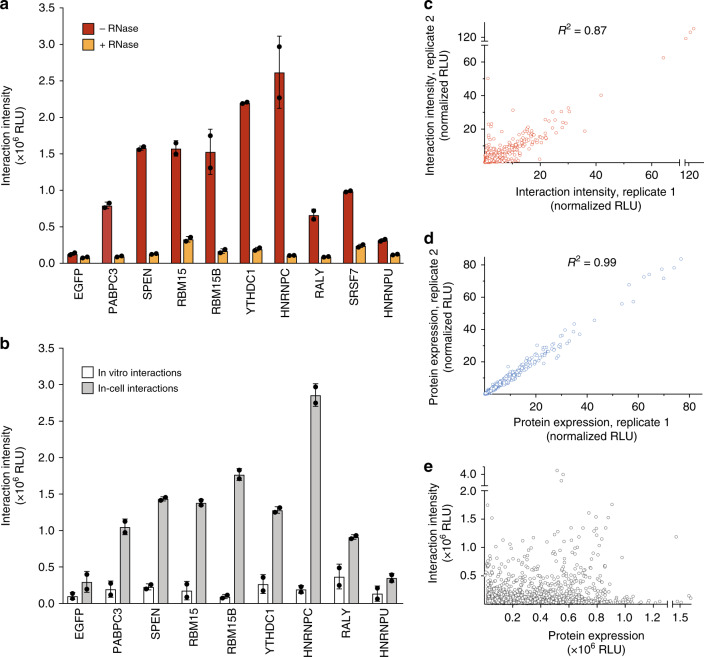
incPRINT measures cellular RNA–protein interactions. **a** Interaction intensities detected between *Xist(A)-MS2* and the indicated factors, with and without RNase treatment. Data from two biological replicates are presented as mean ± s.d.; RLU are relative light units. **b** Interaction intensities between *Xist(A)-MS2* and the indicated factors. In-cell interaction values were measured in a standard incPRINT experiment. In vitro interaction values resulted from separate transfections of the tagged proteins and *Xist(A)-MS2* RNA, which were combined for interaction analyses after the cells were lysed. Data from two biological replicates for each experiment are presented as mean ± s.d.; RLU are relative light units. **c** Scatter plot showing the correlation of the *Xist(A)-MS2* interaction intensities determined by NanoLuc luciferase luminescence from two biological replicates. Shown are median-normalized values. Squared Pearson correlation coefficient is indicated. **d** Scatter plot showing the correlation of FLAG-tagged protein expression levels determined by anti-FLAG ELISA from two biological replicates. Shown are median-normalized values. Squared Pearson correlation coefficient is indicated. **e** Scatter plot showing RNA–protein interaction intensities and protein expression levels. RLU are relative light units.

To establish incPRINT, we performed a series of small-scale experiments using a ∼1-kb conserved region of the lncRNA *Xist* called the A repeat, hereafter referred to as *Xist(A)*^13^. Because several *Xist(A)*-protein interactions have been well established^7^, they served as controls in our initial incPRINT experiments. We engineered a construct to express *Xist(A)-MS2* and assayed the ability of *Xist(A)-MS2* RNA to interact with a selected set of previously identified *Xist*-binding proteins^7,14,15^. The non-discriminatory poly(A)-binding protein PABPC3 was used to control for RNA expression and EGFP (Enhanced Green Fluorescent Protein) was used as a negative control. incPRINT luminescence detected specific interactions of *Xist(A)-MS2* with SPEN, RBM15, RBM15B, YTHDC1, HNRNPC, SRSF7, and RALY, whereas HNRNPU, reported to bind full-length *Xist* but not specifically *Xist(A)*^7^, and EGFP showed basal binding (Fig. 2a). The treatment with RNase abolished the RNA–protein interaction signal measured by luciferase, while the expression of test proteins detected by ELISA remained mostly unchanged (Fig. 2a, Supplementary Fig. 1a), demonstrating that the interactions between the tagged proteins and the luciferase detector were bridged by RNA.

To optimize the number of MS2 stem loops used to tag the tested RNA, *Xist(A)* was fused with two, four, six, ten or 24 MS2 stem loops, and their interactions with a set of control proteins were tested in a small-scale incPRINT experiment. An increase in the luminescence intensity directly correlated with an increased number of MS2 stem loops up to ten stem loops with no marked increase in binding to the EGFP control (Supplementary Fig. 1b). Therefore, in all subsequent incPRINT experiments, the RNAs were tagged with ten MS2 stem loops.

To determine whether the RNA–protein interactions detected by incPRINT indeed occurred in cells or arose only in vitro after cell lysis^16–18^, the luminescence signal from two independent experiments was measured and compared. In the first experiment, *Xist(A)*-*MS2* RNA and FLAG-tagged test proteins were co-transfected in the same cell population as described above. In the second experiment, *Xist(A)-MS2* RNA and FLAG-tagged test proteins were transfected separately in two different cell populations and pooled together only after the cell lysis step, permitting the formation of RNA–protein complexes exclusively in vitro (Supplementary Fig. 1c). We found that interactions were preferentially detected when *Xist(A)-MS2* RNA and the FLAG-tagged proteins were co-transfected (the standard incPRINT condition; Fig. 2b). These results suggest that whenever an interaction signal was detected by incPRINT, it stemmed from the RNA–protein complexes formed in cells, whereas association of RNA–protein complexes post-cell lysis appeared as negligible background under incPRINT-specific conditions (Fig. 2b). Taken together, these experiments establish that incPRINT measures cellular RNA–protein interactions using a luminescence readout.

### High-throughput detection of RNA–protein interactions

To test the scalability of incPRINT for systematic identification of RNA–protein interactions, we interrogated our customized library of ~3000 FLAG-tagged human proteins (including ∼1500 known RBPs^10,11^, ∼1300 transcription factors^12^, and ∼170 chromatin-associated proteins), with *Xist(A)-MS2*. To strengthen the confidence of the incPRINT-identified RNA–protein interactions, all interactions were assayed in biological duplicate, generating two luminescence (RNA–protein interaction intensity) and two ELISA (test protein expression level) values for each tested RNA–protein couple. After filtering out the proteins expressed at insufficient levels (see the ‘Methods’ section), interaction data were analyzed for 2405 proteins. incPRINT’s reproducibility was assessed by calculating the correlation scores of biological duplicates for both the luminescence (Fig. 2c; *R*^2^ = 0.87) and ELISA signals (Fig. 2d; *R*^2^ = 0.99). Notably, no correlation between luminescence and ELISA values was detected, indicating that the interaction intensities were not a mere reflection of the protein expression levels (Fig. 2e). In summary, our data demonstrate that incPRINT is a scalable high-throughput method that reproducibly measures RNA–protein interactions in cell.

### incPRINT identifies proteome of lowly expressed RNAs

**Fig. 3:**
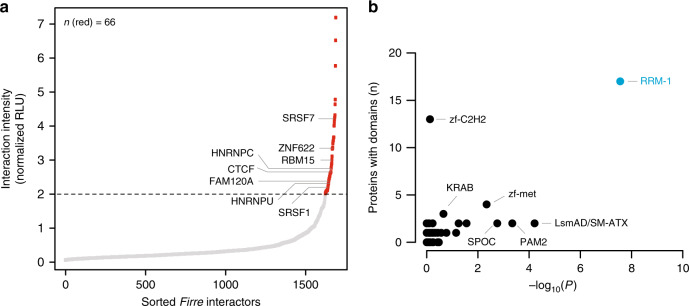
incPRINT identifies *Firre*–interacting proteins. **a** Normalized *Firre-*protein interaction intensities averaged from two biological replicates, sorted in increasing order. The horizontal dotted line represents interaction intensity cutoff used for the classification of *Firre* interactors. Red dots are *Firre-*interacting proteins; gray dots are proteins that do not bind to *Firre*. Proteins either known to bind to *Firre* or those whose interaction was validated in ENCODE eCLIP data^21^ are indicated. See the ‘Methods’ section for data normalization. RLU are relative light units. **b** Plot showing the number of incPRINT *Firre*-interacting partners that contain a particular Pfam domain vs. the −log_10_(*P*) of the domain’s enrichment. *P* values were calculated using the proportion test. Shown in blue are domains represented at least three times in the pulled-down fraction that have a corrected *P* *<* 0.05. RRM-1 refers to the Pfam domain PF00076. For all analyzed Pfam domains, see Supplementary Data 3.

Next, we sought to test if incPRINT can robustly identify proteins interacting with transcripts expressed at low endogenous levels. Identification of proteins associated with low copy number RNAs is generally challenging when using RNA affinity capture-MS approaches due to the typically low efficiency of RNA purifications and the large amount of material required for mass spectrometry. Because *Firre* is a functionally important lncRNA that modulates higher-order nuclear architecture across chromosomes^19^ and is of a rather low endogenous abundance (∼20 molecules per cell based on RNA-Seq data across different mouse tissues), we assessed its RBP-interactome with incPRINT. The full-length *Firre* transcript tagged by *MS2* was expressed ∼40-fold higher than endogenous *FIRRE* in the HEK293T cells used for incPRINT (Supplementary Fig. 2a). As reported for the endogenous transcript^19^, *Firre-MS2* was preferentially localized to the nucleus (Supplementary Fig. 2b). Interrogating our library of ~3000 proteins, incPRINT identified a set of specific proteins as *Firre* interactors (Fig. 3a, red dots; Supplementary Data 1), whereas the majority of the proteins did not interact with *Firre* (Fig. 3a, gray dots; Supplementary Data 1). Importantly, incPRINT identified both known and novel *Firre*-interacting proteins. CTCF and HNRNPU, previously reported by two independent studies to bind *Firre* and to be important for its function^19,20^, were also identified by incPRINT (Fig. 3a). To validate binding of novel *Firre* interactors, we analyzed the ENCODE eCLIP data^21^. The eCLIP data, available for seven incPRINT-identified *Firre*-interacting RBPs, confirmed their binding to *Firre* in the K562 cell line, further validating the incPRINT method (Supplementary Fig. 2c; Supplementary Data 2). Consistent with the role of *Firre* in nuclear organization^19^, a set of *Firre* interactors identified by incPRINT were chromatin-associated proteins including CHD1, POU5F1, JARID2, EPC1, SATB1, MECP2, AEBP2, and CTCF (Supplementary Data 1). Protein domain analyses showed that *Firre*-interacting proteins were significantly enriched for the RNA recognition motif (RRM) (Fig. 3b; Supplementary Data 3).

To determine if RNA overexpression was required for incPRINT to successfully identify proteins binding to RNA with low endogenous levels, a set of proteins was tested with different concentrations of *Firre-MS2* ranging from overexpression as described above to the levels comparable to endogenous *FIRRE* in HEK293T cells (Supplementary Fig. 2d, e). While RNA overexpression resulted in higher interaction scores enabling their better separation from the background scores, the luciferase signal was robustly detectable above background signal when dilutions of *Firre-MS2* were used (Supplementary Fig. 2d). Importantly, this signal was not associated with the test protein expression levels (Supplementary Fig. 2e). Together, these data demonstrate the utility of incPRINT in identifying proteins associated with transcripts expressed at low endogenous levels.

### incPRINT identifies RNA-region-specific interaction partners

**Fig. 4:**
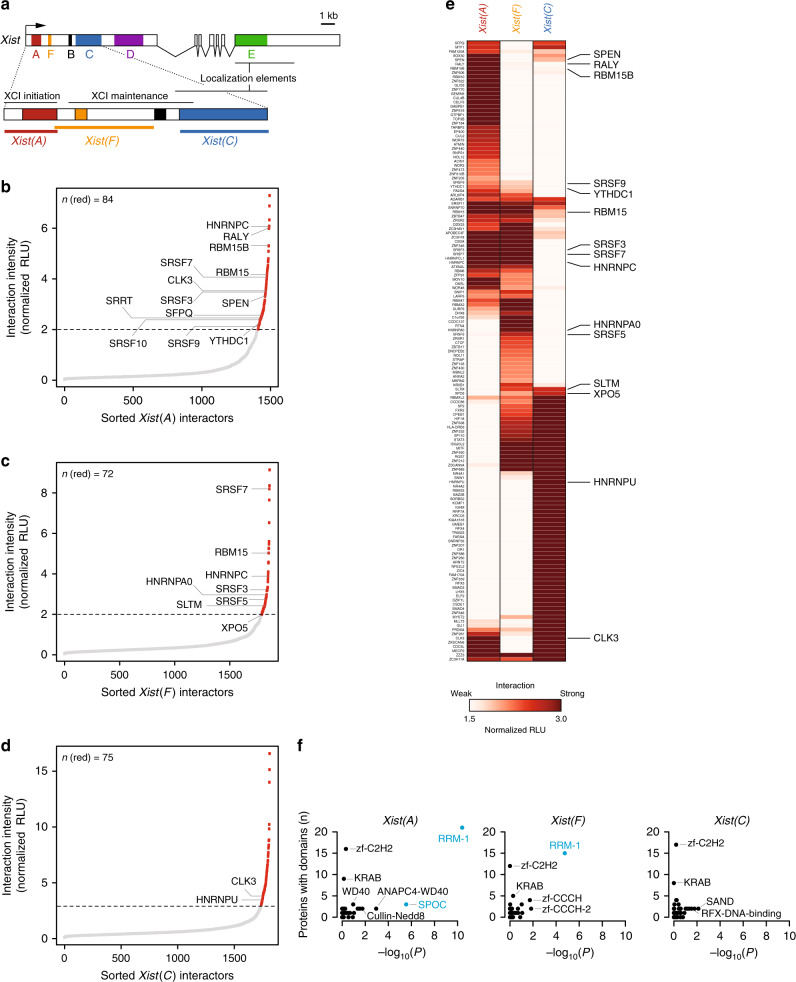
Proteins identified to interact with distinct regions of the lncRNA *Xist*. **a** Schematic representation of the mouse *Xist* transcript and its A- to F-conserved repeat regions. Exons are indicated as boxes, introns as lines. A zoom-in image of the 5′ region of *Xist* shows the positions of *Xist* fragments along the *Xist* transcript. The 0.9-kb, 2-kb, and 1.7-kb fragments for *Xist(A)*, *Xist(F)*, and *Xist(C)*, respectively, used in the incPRINT experiments are delimited by horizontal colored bars. **b** Normalized *Xist(A)-*protein interaction intensities averaged from two biological replicates, sorted in increasing order. The horizontal dotted line represents the interaction intensity cutoff used for the classification of *Xist(A)* interactors. Red dots are *Xist(A)-*interacting proteins; gray dots are proteins that do not bind to *Xist(A)*. Selected proteins known to bind to *Xist* are indicated. See the ‘Methods’ section for data normalization. RLU are relative light units. **c** As in (**b**), for *Xist(F)*. **d** As in (**b**), for *Xist(C)*. **e** Heatmap showing interaction intensities between *Xist(A)*, *Xist(F)*, and *Xist(C)*. Previously detected *Xist*-interacting proteins are indicated on the right. RLU are relative light units. **f** As in Fig. 3b, for *Xist(A)*, *Xist(F)*, and *Xist(C)* interaction partners. SPOC refers to Pfam domain PF07744.

Because many lncRNAs function as modular scaffolds, enabling binding of specific RBPs to discrete RNA domains^1,5^, we sought to test if incPRINT allows the identification of RNA-domain-specific interactions. An ideal proof-of-principle molecule is the lncRNA *Xist*, given its vital role in mammalian X-chromosome inactivation (XCI)^22,23^, and its modular structure and function. The ∼17-kb long *Xist* transcript contains several conserved sequence regions (called repeats A through F) that carry out distinct functions during the XCI process, including initiation of gene silencing (the A repeat), maintenance of the X-inactive state (the F- and B-repeats) and proper chromosomal localization and focal accumulation of *Xist* (the C- and E-repeats)^13,24–32^ (Fig. 4a). Moreover, several independent studies have previously identified and validated a set of functional protein interactions with full-length *Xist*^7,14,15,26,28,33–35^. We sought to apply incPRINT to three conserved regions of mouse *Xist*, i.e., *Xist(A)*, *Xist(F)*, and *Xist(C)* (Fig. 4a). When expressed in HEK293T cells used for incPRINT, each *Xist-MS2* fragment showed a different level of expression compared to endogenous *Xist*, ranging from a ∼60-fold increase for *Xist(A)* to a near-endogenous expression level for *Xist(C)* (Supplementary Fig. 3a). All individual *Xist-MS2* fragments were preferentially localized to the nucleus, similarly to their full-length endogenous counterpart (Supplementary Fig. 3b). Each *Xist* region (i.e., *Xist(A)*, *Xist(F)*, and *Xist(C)*) was interrogated with our library of ~3000 proteins. To compare signals across individual *Xist* regions expressed at different levels (Supplementary Fig. 3c), the interaction scores for each *Xist* region were normalized using the *MS2* RNA binding data (Supplementary Data 4). For normalization, a set of 200 proteins with top ranking luciferase scores in the *MS2* RNA dataset was defined as common binders of all *MS2*-tagged RNAs. The common binders were then identified in each dataset and their median interaction score was calculated for each tested RNA and used to normalize raw luminescence intensities in each dataset (see the ‘Methods’ section). Notably, *MS2* data were not used as a binding specificity control, as many RBPs recognize low complexity RNA motifs^36^ present also within the *MS2* tag, and since protein binding to *MS2* does not exclude a potential functional interaction with a test RNA. Similarly to *Firre*, we found that the majority of proteins did not bind to any of the tested *Xist* fragments (Fig. 4b–d, gray dots; Supplementary Data 4), whereas specific sets of proteins were identified to interact with each individual *Xist* region (Fig. 4b–d, red dots; Supplementary Data 4). Importantly, among the incPRINT-identified *Xist*-interacting proteins, we found well-known interaction partners of *Xist* identified in previous studies to bind the full-length transcript^7,14,15^ (indicated in Fig. 4b–d, Supplementary Table 1). Comparing the sets of incPRINT-identified proteins and their interaction scores for each interrogated *Xist* region (Supplementary Data 4), we found that each *Xist* fragment interacted with a set of proteins specific to the corresponding region, with a minor fraction of RBPs binding to all three *Xist* regions (Fig. 4e). Thus, applying incPRINT to three conserved regions of *Xist* enabled the identification and assignment to specific RNA regions of RBPs previously determined to bind the full-length *Xist* transcript^7,14,15^ (Fig. 4e; known *Xist*-interacting proteins are indicated on the right). For example, incPRINT identified SPEN as a *Xist(A)*-specific interactor (Fig. 4e), confirming previous findings^7,8^. Similarly, RBM15, RBM15B and YTHDC1 were identified by incPRINT to interact specifically with *Xist(A)* and *Xist(F)*, but not *Xist(C)*, confirming their reported binding to the 5′ end of *Xist*^7,9^ (Fig. 4e). Moreover, we identified an *Xist(C)*-specific interaction with HNRNPU (also known as SAF-A) previously shown to be involved in *Xist* localization^7,14,33^ (Fig. 4e, Supplementary Table 1). To validate the RNA-region-specific *Xist*-protein interactions, the ENCODE eCLIP data^21^ available for 14 incPRINT-identified proteins, several of which are novel *Xist*-interacting RPBs, confirmed their binding to *XIST* in the K562 line (Supplementary Fig. 3d**;** Supplementary Data 2), further corroborating the specificity of our method. A functional difference among the protein interactomes of the three *Xist* regions was confirmed by Gene Ontology (GO) term enrichment analyses. Consistent with the differential functions reported for the individual *Xist* regions, *Xist(A)*- and *Xist(F)*-associated proteins were enriched for RBPs involved in RNA processing, while the C-repeat region preferentially interacted with DNA-binding proteins involved in transcriptional regulation (Supplementary Fig. 3e, f). In agreement with the GO analysis, the protein domain analysis demonstrated that *Xist(A)*-interacting proteins were enriched for the SPOC (Spen paralog and ortholog C-terminal) and RRM protein domains, *Xist(F)*-interacting proteins were enriched for the RRM domain and *Xist(C)*-interacting proteins showed no particular enrichment (Fig. 4f; Supplementary Data 3), further highlighting the specificity of incPRINT-identified protein sets for each *Xist* region. In summary, incPRINT successfully retrieved known *Xist*-protein interactions and uncovered novel RBPs. By identifying specific sets of proteins interacting with individual conserved regions of a modular lncRNA, we have demonstrated that incPRINT enables the region-specific assignment of RNA–protein interactions.

### incPRINT identifies functional RNA–protein interactions

**Fig. 5:**
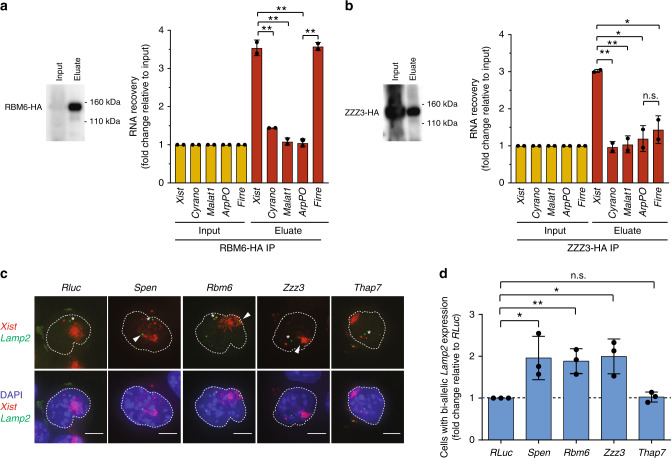
RBM6 and ZZZ3 are required for XCI in vivo. **a** RNA immunoprecipitation (RIP) of the HA-tagged RBM6 protein. Left panel, western blot for RMB6. Right panel, RNA levels of the indicated transcripts in the input and in the immunoprecipitated eluates. All enrichments are normalized to *GAPDH* mRNA and to the input sample. Each RIP experiment was performed on two independent biological replicates. Data are presented as mean ± s.d.; unpaired *t*-tests: **P < 0.01. **b** RNA immunoprecipitation (RIP) of the HA-tagged ZZZ3 protein. Left panel, western blot for ZZZ3. Right panel, RNA levels of the indicated transcripts in the input and in the immunoprecipitated eluates. All enrichments are normalized to *GAPDH* mRNA and to the input sample as described in the ‘Methods’ section. Each RIP experiment was performed twice on independent biological replicates. Data are presented as mean ± s.d.; unpaired *t*-tests: **P < 0.01; *P < 0.05, not significant (ns). Full blots are provided as a Source Data file. **c** Representative RNA FISH images of *Xist*-induced cells upon depletion of the indicated proteins. *Xist* is shown in red and the X-linked gene *Lamp2* in green. The dashed line delineates cell nuclei. Asterisks indicate *Lamp2* expression from the active X chromosome. Arrowheads indicate *Lamp2* expression from the inactive X chromosome that escapes XCI. Scale bars, 5 μm. **d** Quantification of cells with bi-allelic *Lamp2* expression assessed with RNA FISH and expressed as fold ratio over *RLuc* control. Data from three independent experiments are represented as mean ± s.d.; Student’s *t*-tests: **P < 0.01; *P < 0.05; not significant (ns). Dashed line delineates the level of *RLuc*.

Because *Xist* has a well-characterized cellular function in gene silencing during XCI, we sought to test if some of the *Xist*-protein interactions uncovered using incPRINT are functionally relevant. The focus was on the ZZZ3 protein, which interacts with all three tested *Xist* regions, and RBM6, which exhibits more specific binding to the *Xist(A)* and *Xist(F)* regions (Fig. 4e). First, we confirmed the interaction of *Xist* with RBM6 and ZZZ3 under endogenous conditions by testing *Xist* co-precipitation with both proteins in mouse embryonic stem (ES) cells. The proteins were HA-tagged in the polymorphic TX1072 ES cell line that enables doxycycline-induced *Xist* expression, triggering XCI in the absence of differentiation^31,37–39^. RNA immunoprecipitation (RIP) after doxycycline induction of *Xist* and UV-cross-linking of cells followed by qRT-PCR analyses identified a significant enrichment of the *Xist* transcript with RBM6 and ZZZ3 proteins, confirming their interaction in vivo (Fig. 5a, b). Notably, incPRINT also identified RBM6 as interacting with *Firre*. RIP qRT-PCR detected a specific interaction of *Firre* with RBM6 but not with ZZZ3, thus confirming the *Firre*-RBM6 binding under endogenous conditions and further validating our incPRINT results (Fig. 5a, b).

Next, to test if RBM6 and ZZZ3 have an impact on XCI, we used single-cell RNA fluorescent in situ hybridization (RNA FISH) to assess the expression of endogenous *Lamp2*, an X-linked gene that is normally silenced during XCI initiation^40^. Upon doxycycline-induced *Xist* expression, depletion of *Rbm6*, *Zzz3*, and positive control *Spen* (Supplementary Fig. 4a, b) led to reduced silencing of *Lamp2*, whereas its XCI-induced mono-allelic expression remained unaltered upon depletion of *Thap7*, which did not interact with *Xist* and was used as a negative control (Fig. 5c, d; Supplementary Fig. 4c; Supplementary Table 2). In the absence of *Xist* (-doxycycline conditions), *Lamp2* expression remained unchanged upon depletion of the proteins above (Supplementary Fig. 4d; Supplementary Table 2). Importantly, the defects in X chromosome silencing were not triggered by altered expression of *Xist/Tsix* upon depletion of the individual proteins (Supplementary Fig. 4e). In summary, the identification of functionally important interactors among the incPRINT-identified RBPs demonstrates incPRINT’s potential for discovery.

## Discussion

Several methods have been developed to comprehensively define the RNA-binding protein repertoire of all cellular RNAs^10,11,41–45^. To identify the protein interactomes of individual RNAs of interest, we report here incPRINT, a reliable RNA-centric technology which we show uncovers both known and previously unknown RNA–protein interactions. incPRINT successfully overcomes two major obstacles in determining the proteomes of specific RNAs: first, incPRINT enables the identification of proteins associated with RNAs expressed at low endogenous levels, common for many lncRNAs and, second, incPRINT allows the detection of RNA-region-specific protein interactions and the assignment of protein binding to defined regions of a full-length transcript. This is particularly advantageous for defining the RNA-region-specific binding proteome of large modular RNAs with multiple functional regions, such as the ∼17-kb-long *Xist* transcript. While other RNA-centric methods such as dChIRP have allowed the validation of already known RNA-region-specific protein interactions^6^, incPRINT enables their de novo identification. Considering this feature of incPRINT, one could envision a higher-resolution mapping of RBP binding along a transcript of interest by applying a low- or medium-throughput-scale incPRINT to shorter RNA fragments or their mutated variants.

Several key aspects of incPRINT distinguish it from the RNA-centric methods currently employed for the identification of RNA–protein interactions. First, incPRINT does not rely on probe-based capture and direct RNA affinity purification, which generally exhibits a low efficiency and thus requires large amounts of material. Because it uses a sensitive luciferase detector, incPRINT is not limited by RNA low copy number and can be applied to any RNA of interest. Second, the quantitative nature of the luciferase detector used in incPRINT makes it suitable for various custom applications and offers the possibility of structure/function analyses of mutated or disordered RNA–protein interactions and their relative effects on binding affinities. Third, the ability of incPRINT to measure cellular RNA–protein interactions one by one, independently of the cell’s physiological state, is particularly relevant for defining RNA-bound proteomes of transcripts that display a dynamic expression range during development and/or cellular differentiation, as observed with *Xist* throughout different stages of XCI^7^. Finally, incPRINT is flexible in its throughput: we employed here a customized library of ~ 3000 human test proteins comprising together ~1/7 of the human proteome and representing the majority of all known RBPs, transcription factors and chromatin modifiers. Using this protein library, we discovered functional interactors that had evaded detection with other methods; however, the library can conveniently be expanded to include a range of additional proteins, or reduced and customized to fit the experimental design and need.

While incPRINT enables fast, systematic and quantitative identification of RNA–protein interactions, it is a binding assay that does not consider the developmental context or timing of these interactions. However, this potential limitation did not hinder the identification of *Xist*-protein interactions in a heterologous cell line. Because incPRINT is based on ectopic expression of the test protein and RNA components, it is possible that a fraction of the identified interactions will be false positives. While such interactions cannot be excluded, the number of highly specific interactors for each interrogated RNA corresponding to 2–3% of all screened proteins and the validation of a set of interactions by orthogonal methods suggest that the rate of false positive binding identified by incPRINT is rather low. Furthermore, whereas all interactions validated in this study were direct, the design of the method does not make it possible to distinguish between direct and indirect RNA–protein interactions and additional assays are required to stipulate direct RNA binding. In summary, applying the incPRINT method to other noncoding or coding RNAs of various structural complexities will facilitate the discovery of the precise mechanisms by which they exert their cellular functions through the identification of their binding proteins.

## Methods

### Cell culture

To generate the stable monoclonal HEK293T cell line (gift from the Raphael Margueron laboratory, Institut Curie) expressing NanoLuc-MS2CP fusion protein, the *NanoLuc-MS2CP* expression plasmid was transfected using polyethylenimine (PEI) followed by puromycin selection. The expression of NanoLuc-MS2CP was verified by measuring luciferase activity. The cells were maintained in DMEM (Gibco 12007559) containing 10% fetal bovine serum (Gibco 11573397) and 1% penicillin/streptomycin (Gibco 15140122).

The female hybrid mouse TX1072 ES cell line^37^ (Edith Heard laboratory, Institut Curie) that harbors a doxycycline-responsive promoter controlling *Xist* expression on the B6 X chromosome and was derived from a cross of a TX/TXR26^rtTA/rtTA^ female^38^ with a male *Mus musculus castaneus* was used. ES cells were cultured in high-glucose DMEM (Sigma) supplemented with 15% fetal calf serum (Eurobio, S59341-1307), 0.1 mM β-mercaptoethanol, 1000 U/mL leukemia inhibitory factor (LIF, Chemicon), and 2i (3 μM GSK3 inhibitor CT-99021, and 1 μM MEK inhibitor PD0325901). *Xist* expression was induced with doxycycline (Sigma) for 24 h for RNA FISH analysis.

### Generation of the HA-tagged RBM6 and ZZZ3 ES cell lines

The TX1072 ES cell line^37^ was used to generate HA-tagged RBM6 and ZZZ3 cell lines. The HA tag was introduced at the C-terminus of RBM6 and ZZZ3. The tag flanked by two ∼600 nt homology arms was cloned into a pBR322 vector. TX1072 ES cells were transfected using Lipofectamine 2000, with the RBM6 or ZZZ3 HA-targeting vectors together with the pX459 vector containing a gRNA sequence targeting each of the proteins, promoting homologous recombination. Transfected clones were selected with 0.4 μg/mL of puromycin. Clones were genotyped for integration at the correct genomic location by PCR and DNA sequencing to ensure no deletions or mutations in the protein-coding sequence. All relevant oligonucleotide sequences are listed in Supplementary Data 5.

### esiRNAs

Gene knockdown was achieved using esiRNAs^46^ (Eupheria Biotech) directed against *Rbm6* and *Zzz3*, luciferase and *Thap7* as negative controls and *Spen* as a positive control. esiRNA transfections into TX1072 ES cells were conducted using 0.1 μg esiRNAs and Lipofectamine 2000 Transfection Reagent (Thermo Fisher Scientific), in Optimem I reduced serum medium (Thermo Ficher Scientific). Doxycycline-mediated *Xist* expression was initiated 48 h post-transfection, and cells were harvested 24 h post-doxycycline induction of *Xist*. The same experimental setup was used for RNA-FISH, RT-qPCR and RIP experiments. To assess esiRNA knockdown efficiencies, 50 ng of total RNA were reverse-transcribed using the SuperScript IV kit (Invitrogen) followed by qPCR using SYBRgreen (Applied biosystems). *Arppo* mRNA was used to normalize RNA levels between samples. All relevant oligonucleotides are listed in Supplementary Data 5.

### Generation of incPRINT expression constructs

To generate the luciferase-MS2CP expression vector, NanoLuc luciferase was amplified from the pNL1.1 plasmid (Promega) and cloned into the pCi-MS2 vector^47^ using PstI and BamHI restriction sites. A puromycin resistance gene was added to the plasmid using PvtI restriction site. To remove the FLAG tag present in the original plasmid, a stop codon was introduced between the MS2CP and the FLAG tag by site-directed mutagenesis (Agilent).

To generate customized RNA-10xMS2 constructs, the pCDNA3.1 plasmid (Thermo Fisher) was modified. First, additional restriction sites (BstBI, AgeI, ClaI, AscI, PacI, BglII, and SrfI) were incorporated between the KpnI and BamHI sites. Second, MS2 stem loops were inserted between the BamHI and BglII sites. *Xist(A)*, *(F)*, and *(C)* fragments were PCR-amplified from BAC 399K20 (covering chrX:100,578,985–100,773,006, mm9 genome assembly), and cloned upstream of the MS2 stem loops by Gibson assembly (New England Biolabs). Primers used for *Xist* fragments amplification and cloning are indicated in Supplementary Data 5.

### Human FLAG-tagged protein library

The transcription factor collection has been previously described^12^. The collection of human RBPs and chromatin-associated proteins were cloned with Gateway recombination from the human ORFeome 5.1 (http://horfdb.dfci.harvard.edu/hv5/index.php) into a mammalian expression vector with a C-terminal 3xFLAG-V5 tag. The expression clones were verified by restriction enzyme digestion.

### incPRINT

384-well plates (Greiner Bio-One 781074) were coated overnight with the anti-FLAG M2 antibody (F1804, Sigma; 10 μg/ml in 1x PBS) and blocked for two hours at room temperature in 1% BSA, 5% sucrose, 0.5% Tween 20 in 1x PBS. Plasmids encoding RNA-MS2 and 3xFLAG-tagged test proteins were co-transfected into a 293T stable cell line expressing the NanoLuc luciferase fused to MS2CP. The day before transfection, cells were seeded in 96-well plates (30,000 cells per well). Co-transfections were performed using 12 µg/ml (300 ng of each plasmid per well). To achieve different expression levels of *Firre-MS2*, 30 ng (1:10), and 6 ng (1:50) of the *Firre-MS2* encoding vector were used for transfections. Two days after transfection, cells were washed twice with ice-cold 1x PBS and lysed in ice-cold RQ1-HENG buffer (20 mM HEPES-KOH [pH 7.9], 150 mM NaCl, 10 mM MgCl_2_, 1 mM CaCl_2_, 5% glycerol, 1% Triton X-100, supplemented with protease inhibitors) containing 30 U/ml of RQ1 DNase (Promega M6101). After lysis (10 min, 4 °C) and DNase incubation (30 min, 37 °C), the lysates were transferred to 384-well plates coated with anti-FLAG M2 antibody (F1804, Sigma; 10 μg/ml in 1x PBS). Following a three-hour incubation at 4 °C, plates were washed seven times with RQ1-HENG buffer. Furimazine substrate (Promega) was added to the plates and luminescence in each well was measured with a plate reader (Biotek Synergy Neo). Following luminescence measurement, HRP-conjugated anti-FLAG antibody (ab1238, Abcam) in ELISA buffer (1x PBS, 1% goat serum, 1% Tween 20) was added to each well. After 30 min of incubation at room temperature, plates were washed with 1x PBS, 0.05% Tween 20, HRP substrates (Pierce) were added and ELISA signals were detected with the plate reader. For the RNase treatment experiment, RNA–protein interactions were first measured as described above. Fifty microliters of RNase buffer containing 50 mM Tris-HCl pH 8.0, 10 mM EDTA, +/−100 μg/ml RNase A (Qiagen) were added to the samples and incubated for 30 min at 37 °C, followed by three washes with RQ1-HENG buffer, before proceeding with luminescence measurements. All 96- and 384-plate washes were performed with a plate washer (Biotek EL406).

### incPRINT data analysis and normalization

For each studied RNA (i.e., *Firre-MS2*, *Xist(A)-MS2*, *Xist(F)-MS2*, *Xist(C)-MS2*), all FLAG-tagged proteins were tested in duplicates achieved by independent transfections. After measuring the NanoLuc and ELISA luminescence, log_2_-transformed ELISA values were binned to assess the distribution of the FLAG-tagged protein expression. Proteins with the lowest ELISA signals were filtered out (status 2 in Supplementary Data 1 and 4). Interaction values between a test *RNA-MS2* and a test protein were defined as the average between the two luciferase luminescence replicates. To ensure the robustness of the interaction analysis, luciferase replicates that showed the highest discrepancy (average/s.d. <1.5, unless both duplicates showed a high interaction score) were removed from the dataset (status 3 in Supplementary Data 1 and 4).

To compare the interaction intensities among individual *RNA-MS2* transcripts, raw luminescence intensities were normalized. A set of proteins exhibiting a luminescence signal with *MS2* was defined as common binders, assumed to interact with all tested *RNA-MS2* transcripts. For each tested *RNA-MS2* transcript, the median interaction scores of this set of common binders was calculated and used to normalize all raw luminescence intensities measured for the corresponding *RNA-MS2*. The comparison between *Xist(A)-MS2*, *Xist(F)-MS2*, and *Xist(C)-MS2* interactors, includes all test proteins showing interaction with at least one test RNA, and expressed in all three assays.

### Protein domain analysis

A list of the Pfam domains of each protein was downloaded from the Ensembl site. Enrichment of each domain in the set of interactors of a particular RNA vs. the protein library was calculated using a proportion test.

### RNA FISH

*Xist* expression was induced in undifferentiated ES cells by addition of 1 μg/mL of doxycycline to the culture medium 48 h post-transfection. Twenty-four hours upon *Xist* induction, cells were harvested using TrypLE Express Enzyme (Thermo Fisher Scientific), washed with 1x PBS, and adsorbed onto Poly-L-Lysine (Sigma)-coated glass coverslips for 10 min. Fixation was performed with 3% paraformaldehyde for 10 min at room temperature, followed by permeabilization in 1x PBS containing 0.5% of Triton X-100 and 2 mM of vanadyl-ribonucleoside complex (New England Biolabs), for 5 min at 4 °C. Coverslips were washed three times in 70% ethanol, and preserved at −20 °C in 70% ethanol. Prior to hybridization, coverslips were dehydrated with increasing concentrations of ethanol (80%, 95%, 100% twice, 5 min each), and air-dried. Transcription of the X-linked gene Lamp2 was detected with a BAC spanning its genomic region (RP24-173A8). The *Lamp2* probe was labeled with dUTP-Spectrum Green (Enzo, Life Sciences) by nick translation (Abbot). The probes were precipitated with ethanol, resuspended in formamide at 37 °C, denatured at 75 °C for 10 min, and competed with mouse Cot1 DNA (Thermo Fisher) for 1–2 h at 37 °C. *Xist* was detected with a dUTP-Spectrum Red (Enzo, Life Sciences) nick translation probe from a plasmid spanning its genomic region^48^. The *Xist* probe was prepared as described above for the *Lamp2* probe, except for the competition step, which was not performed. Probes were mixed and co-hybridized in FISH hybridization buffer (50% formamide, 20% dextran sulfate, 2x SSC, 1 μg/μL BSA (New England Biolabs), 10 mM Vanadyl-ribonucleoside) overnight at 37 °C. Coverslips were washed 3 × 6 min in 50% formamide in 2x SSC, pH 7.2, at 42 °C, followed by two 5-min washes in 2x SSC at 42 °C. Nuclei were counterstained with 0.2 mg/mL of 4′,6-Diamidine-2′-phenylindole dihydrochloride (DAPI) in 2x SSC for 3 min at room temperature, and mounted onto glass slides using VectaShield mounting medium. Images were acquired using the wide-field DeltaVision Core microscope (Applied Precision) and the inverted confocal Spinning Disk Roper/Nikon-FRAP microscope. 3D image stacks were analyzed with ImageJ.

### Western blot analysis

To assess knockdown efficiency at the protein level, TX1072 ES cells were transfected with 2.5 μg of esiRNAs for *RLuc* (as a negative control), *Rbm6* and *Zzz3*. Whole cell extracts were prepared with RIPA buffer (50 mM Tris-HCl pH 8–8.5, 150 mM NaCl, 1% Triton X-100, 0.5% sodium deoxycholate, 0.1% SDS) with freshly added 1x protease inhibitor (Roche). Western blot analysis was performed with antibodies detecting RBM6 (HPA026272, Sigma; 1:250), ZZZ3 (SAB4501106, Sigma; 1:1000) and Tubulin (CP06 mouse monoclonal antibody (DM1A), Merck Millipore; 1:3000). Full blots are provided as a Source Data file.

### RNA immunoprecipitation (RIP)

RIP experiments were performed using corresponding HA-tagged RBM6 and ZZZ3 TX1072 cell lines. Approximately 15 million cells were treated with doxycycline (1 µg/ml) for 16 h. Cells were washed once with ice-cold 1x PBS and UV-crosslinked using 800 mJ/cm^2^, at 254 nm (Stratalinker, Stratagene). Cells were then collected, pelleted and lysed for 10 min on ice in 200 µl of RIPA buffer containing protease inhibitors (Roche) and RNAsin (Invitrogen). Cell lysates were sonicated using a Bioruptor (Diagenode) (6 cycles; 15 s on, 30 s off), and centrifuged for 10 min at 15,000 × *g*. Supernatant were collected and diluted in RQ1-HENG buffer (to 1.2 ml). Fifty microliters of anti-HA bead slurry (Pierce, 88836) was washed twice with 1 ml of RQ1-HENG buffer and incubated with the cell lysate for 2 h at 4 °C. Following the incubation, beads were washed twice with TNM600 buffer (50 mM Tris-HCl [pH 7.8], 0.6 M NaCl, 1.5 mM MgCl_2_, 0.1% NP40), and twice with TNM100 buffer (50 mM Tris-HCl [pH 7.8], 0.1 M NaCl, 1.5 mM MgCl_2_, 0.1% NP40). Elution was performed in 50 µl Laemmli buffer (2 min, 90 °C), RNA was extracted (Trizol-Chloroform) and treated with TURBO DNase (Ambion). Two hundred and fifty nanograms of RNA were reverse-transcribed using the SuperScript IV kit (Invitrogen) followed by qPCR using SYBRgreen (Applied biosystems). *Gapdh* was used as a reference gene to normalize RNA levels between samples. All relevant oligonucleotides are listed in Supplementary Data 5.

### Nuclear/cytoplasmic fractionation and calculation of RNA expression

Two days after the RNA-MS2 transfections, HEK293T cells were centrifuged (5 min, 100 × *g*, 4 °C), washed with 1x PBS, resuspended in 100 μl of buffer A (10 mM HEPES pH 7.9, 5 mM MgCl_2_, 250 mM sucrose, protease inhibitors), incubated for 10 min on ice, and centrifuged again (10 min, 2350 × *g*, 4 °C). The supernatant was collected (cytosolic fraction), and the pellet was resuspended in 100 μl of buffer B (25 mM HEPES pH 7.9, 20% glycerol, 1.5 mM MgCl_2_, 0.1 mM EDTA, 0.7 M NaCl, protease inhibitors). After sonication (Bioruptor, Diagenode; 6 cycles; 15 s on, 30 s off) and centrifugation (21,000 × *g*, 15 min, 4 °C), the supernatant was collected (nuclear fraction). RNA was extracted from both fractions, and treated with Turbo DNase (Ambion). Hundred nanograms of RNA were reverse-transcribed using the SuperScript IV kit (Invitrogen) followed by qPCR analysis using SYBRgreen (Applied Biosystems). *β-actin* was used as a reference gene. Nuclear/cytoplasmic ratios were calculated for every tested transcript. All relevant oligonucleotides are listed in Supplementary Data 5.

### Reporting summary

Further information on research design is available in the Nature Research Reporting Summary linked to this article.

## Supplementary information

Supplementary Information

Reporting Summary

Description of Additional Supplementary Files

Supplementary Data 1

Supplementary Data 2

Supplementary Data 3

Supplementary Data 4

Supplementary Data 5

## Data Availability

A reporting summary for this article is available as a Supplementary Information file. The source data underlying Fig. 5a, b and Supplementary Fig. 1b are provided as a Source Data file. All data generated and analyzed during this study are included in this article and its Supplementary Information file. All data is available from the corresponding author upon reasonable request.

## Source Data

Source Data
