# Author Correction: In-cell identification and measurement of RNA-protein interactions

**DOI:** 10.1038/s41467-020-17282-6

**Published:** 2020-07-08

**Authors:** Antoine Graindorge, Inês Pinheiro, Anna Nawrocka, Allison C. Mallory, Peter Tsvetkov, Noa Gil, Carlo Carolis, Frank Buchholz, Igor Ulitsky, Edith Heard, Mikko Taipale, Alena Shkumatava

**Affiliations:** 1 Institut Curie, PSL Research University, CNRS UMR3215, INSERM U934, Paris, 75005 France; 2 Whitehead Institute for Biomedical Research, Cambridge, MA 02142 USA; 3 Department of Biological Regulation, Weizmann Institute of Science, Rehovot, 76100 Israel; 4 Biomolecular Screening and Protein Technologies Unit, Centre de Regulació Genòmica (CRG), The Barcelona Institute of Science and Technology, Barcelona, Spain; 5 Medical System Biology, UCC, Medical Faculty Carl Gustav Carus, TU Dresden, Dresden, Germany; 6 Max Planck Institute for Molecular Cell Biology and Genetics, Dresden, Germany; 7 Donnelly Centre for Cellular and Biomolecular Research, Department of Molecular Genetics, Toronto, Canada

**Keywords:** RNA, Non-coding RNAs

Correction to: *Nature Communications* 10.1038/s41467-019-13235-w, published online 22 November 2019.

The original version of this Article omitted the following from the Acknowledgements: A.G. was supported by the ARC postdoctoral fellowship (20131200567), short-term EMBO (ESTF324-2015) and Company of Biologists travel (JCSTF-150421) fellowships, and LabEx DEEP (ANR-11-LABX-0044, ANR-10-IDEX-0001-02). This has now been corrected in both the PDF and HTML versions of the Article.

