# Supplementary material for: In-cell identification and measurement of RNA-protein interactions
Source: Nat Commun. 2019 Nov 22;10:5317. doi: 10.1038/s41467-019-13235-w (PMC6876571; doi:10.1038/s41467-019-13235-w)
Supplement: Supplementary file 1 — Supplementary Information [file 41467_2019_13235_MOESM1_ESM.pdf]

SUPPLEMENTARY INFORMATION

In-Cell Identification and Measurement of RNA-Protein Interactions

Graindorge et al.

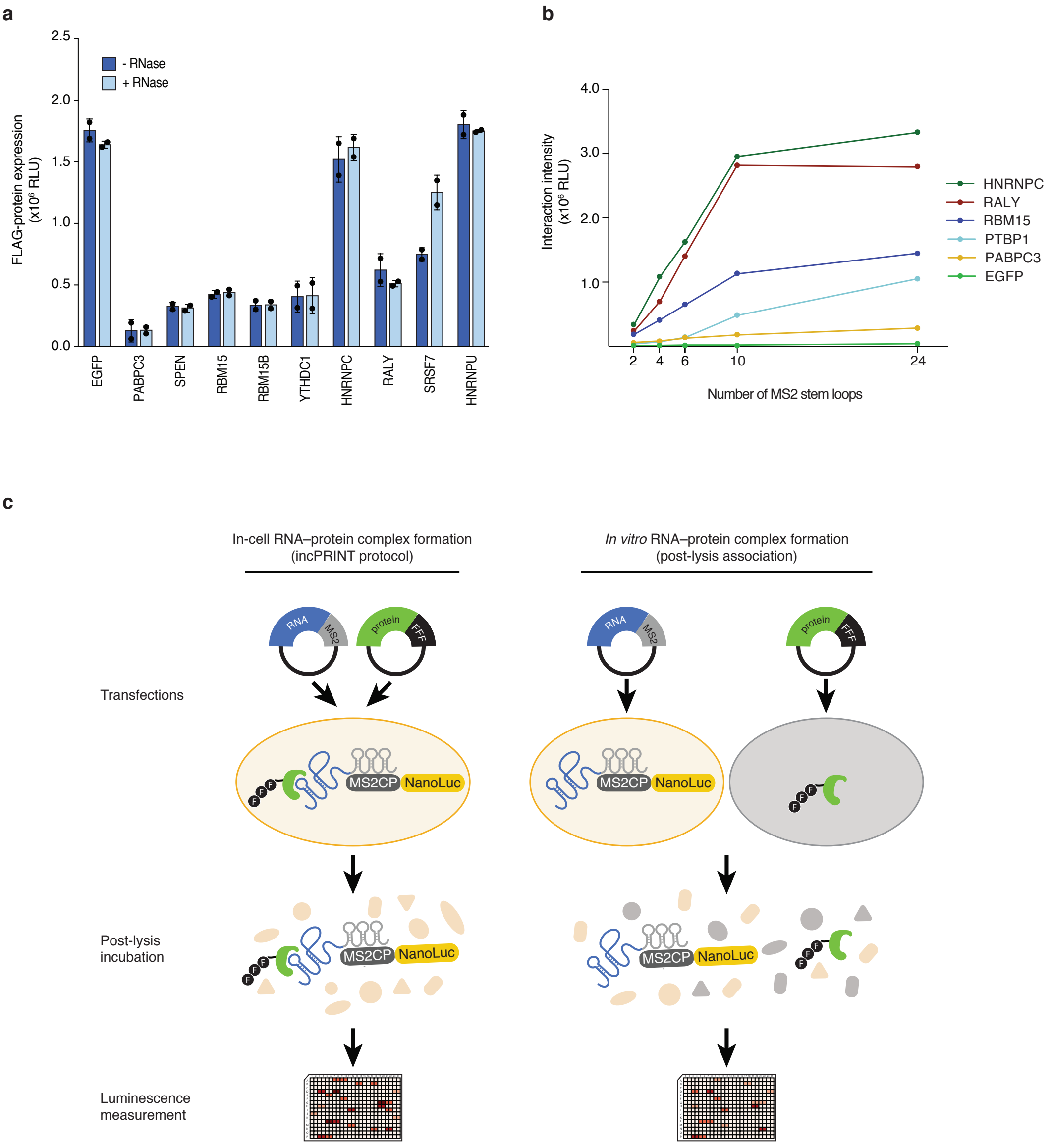

Supplementary Figure 1: incPRINT extended set-up. (a) Expression levels of the indicated FLAG-tagged proteins measured by ELISA, with and without RNase treatment. Data from two biological replicates are presented as mean ± s.d. RLU are Relative Light Units. (b) Changes in interaction intensities between *Xist(A)-MS2* and the indicated factors depending on the number of MS2 stem loops used to tag *Xist(A)*. RLU are Relative Light Units. (c) Workflow of the experiment testing in-cell vs. *in vitro* formation of RNA-protein complexes. In the *in-cell* condition (left panel), *Xist(A)-MS2* and the test protein were co-transfected in the same cell population, and 48 hours after transfection the cells were lysed and lysates were incubated in anti-FLAG-coated plates. In the *in vitro* condition (right panel), *Xist(A)-MS2* and the test protein were transfected in separate cell populations, and 48 hours following transfection the cells were lysed and lysates were pooled and incubated in anti-FLAG-coated 384-well plates. Washes and Luciferase luminescence detection done as in Fig. 1.

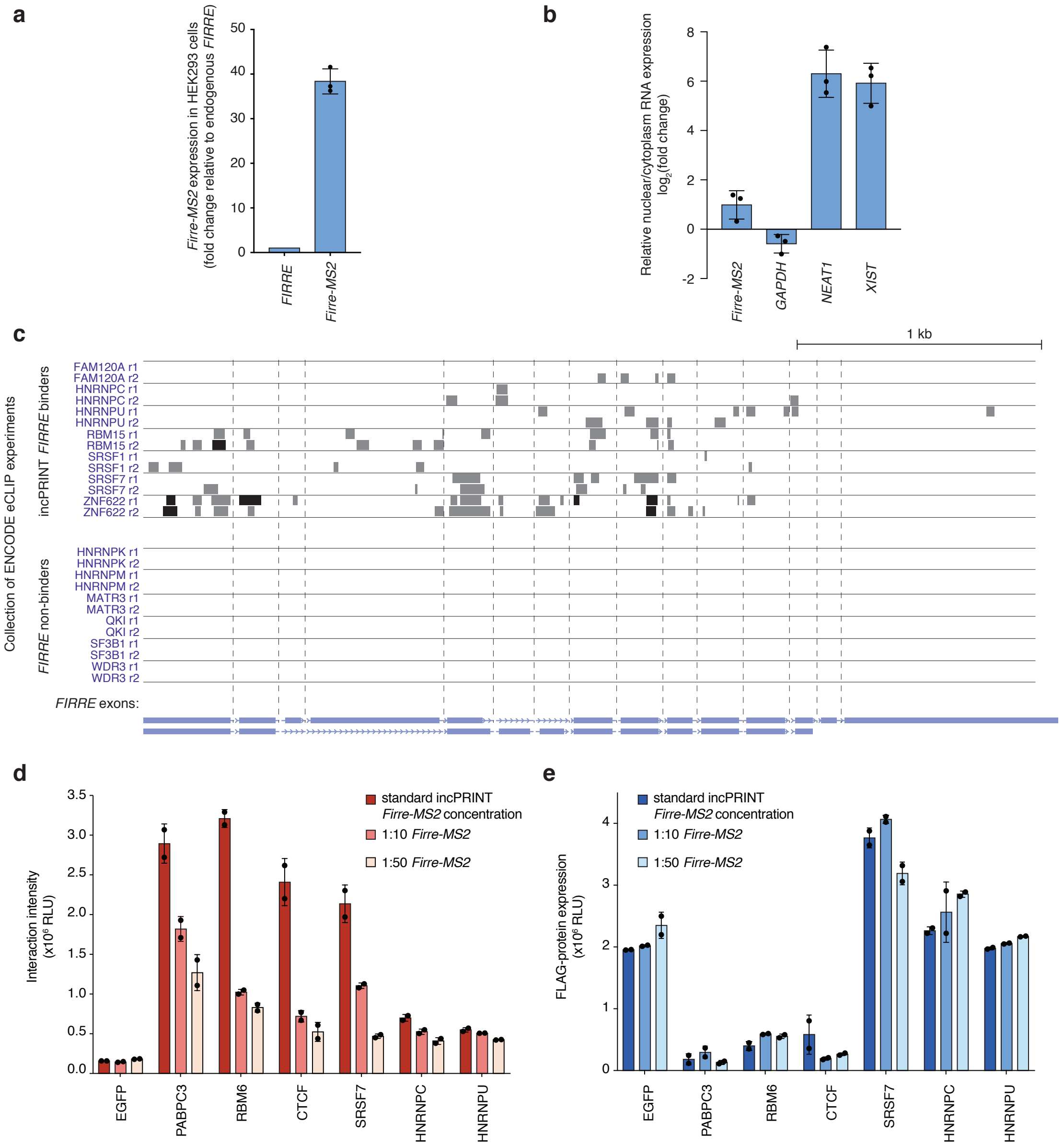

Supplementary Figure 2: *Firre-MS2* expression and validation of *Firre* interactions by eCLIP. (a) qRT-PCR analysis of *Firre-MS2* and endogenous *FIRRE* levels in HEK293 cells. *GAPDH* was used as a reference gene. Data from three independent experiments are represented as mean ± s.d. (b) qRT-PCR analyses of *Firre-MS2* levels in the nuclear and cytoplasm fractions of HEK293 cells. *GAPDH* was used as a reference gene. Data from three independent experiments are represented as mean ± s.d. (c) ENCODE eCLIP data[22] showing binding of the indicated proteins to *FIRRE* in K562 cells. Peaks are as called by the ENCODE project: black boxes indicate peaks with enrichment ≥4 and significance <0.001; gray boxes are all other peaks reported by ENCODE. A set of abundant nuclear proteins with no or substantially less binding to *FIRRE* is shown for comparison. Replicate (r). (d) Interaction intensities detected between the indicated proteins and *Firre-MS2* expressed at different levels, whereas the 1:50 dilution corresponds to the endogenous *FIRRE* expression levels in HEK293T cells. Data from two biological replicates are presented as mean ± s.d.; RLU are Relative Light Units. (e) Expression levels of the indicated FLAG-tagged proteins measured by ELISA. Standard incPRINT protein concentration was used for all tested conditions. Data from two biological replicates are presented as mean ± s.d.; RLU are Relative Light Units.

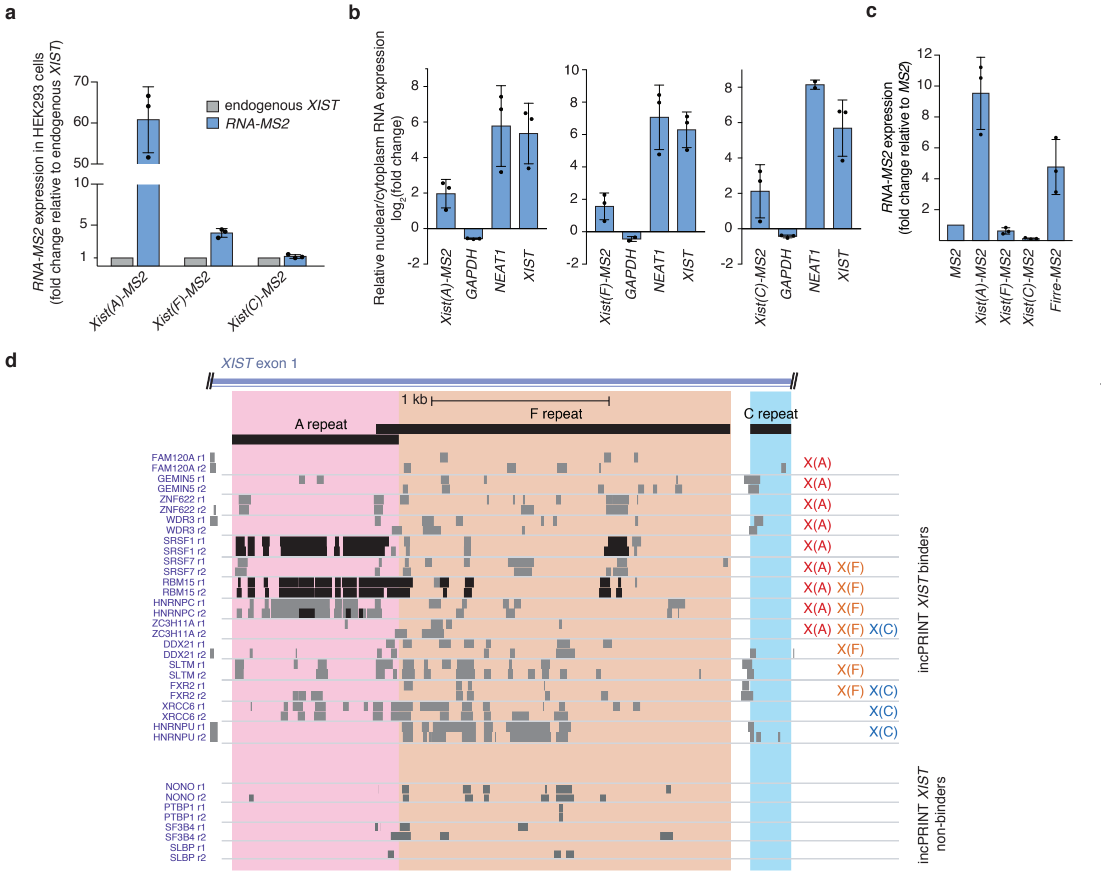

e

GO #	GO terms (Molecular Function)	Xist(A) #proteins (/ 79)	Xist(A) P-value	Xist(F) #proteins (/ 64)	Xist(F) P-value	Xist(C) #proteins (/ 67)	Xist(C) P-value
0000166	Nucleotide binding	24	2.14E-04	16	0.076	5	0.862
0044822	Poly(A) RNA binding	50	3.53E-08	41	1.64E-04	18	0.832
0003723	RNA binding	19	2.15E-03	16	3.65E-03	4	0.986
0003676	Nucleic acid binding	36	8.03E-07	23	5.97E-06	15	0.973
0003677	DNA binding	26	0.797	15	0.994	37	1.22E-03

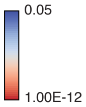

f

GO #	GO terms (Biological Processes)	Xist(A) #proteins (/ 73)	Xist(A) P-value	Xist(F) #proteins (/ 59)	Xist(F) P-value	Xist(C) #proteins (/ 63)	Xist(C) P-value
0048025	Negative regulation of mRNA splicing, via spliceosome	6	2.78E-04	1	1	0	n.d
0048024	Regulation of mRNA splicing, via spliceosome	4	6.04E-03	1	1	0	n.d
0006369	Termination of RNA polymerase II transcription	8	7.98E-04	5	0.037	1	1
0031124	mRNA 3'-end processing	7	1.11E-03	5	0.016	1	1
0006397	mRNA processing	13	6.79E-05	8	0.015	0	n.d
0006405	RNA export from nucleus	7	3.24E-04	5	7.45E-03	1	1
0006406	mRNA export from nucleus	9	1.23E-04	6	8.14E-03	1	1
0008380	RNA splicing	12	3.90E-04	10	1.22E-03	3	0.803
0000398	mRNA splicing, via spliceosome	20	2.62E-07	12	2.23E-03	4	0.840
0006351	Transcription, DNA-templated	25	0.949	19	0.968	38	3.11E-03

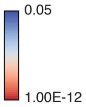

Supplementary Figure 3: Expression of individual *Xist-MS2* regions and validation of *Xist* interactions by eCLIP. (a) qRT-PCR analysis of *Xist(A)-MS2, Xist(F)-MS2, Xist(C)-MS2* and endogenous *XIST* levels in HEK293 cells. *GAPDH* was used as a reference gene. Data from three independent experiments are represented as mean ± s.d. (b) qRT-PCR analyses of *Xist(A)-MS2, Xist(F)-MS2, Xist(C)-MS2* levels in the nuclear and cytoplasm fractions of HEK293T cells. *β-actin* was used as a reference gene. Data from three independent experiments are represented as mean ± s.d. (c) qRT-PCR analyses of the levels of all incPRINT-tested RNAs in HEK293 cells. *GAPDH* was used as a reference gene. Data from three independent experiments are represented as mean ± s.d. (d) Binding of indicated proteins to *XIST* in ENCODE eCLIP data from K562 cells[22]. As defined by the ENCODE project, black boxes

indicate peaks with enrichment ≥4 and significance <0.001 and gray boxes are all other peaks reported by ENCODE. A set of abundant nuclear proteins with no or substantially less binding to *XIST* is also shown for comparison. Replicate (r). (e) Molecular function GO terms enriched for *Xist* region-specific interactors. Background reference population is the full set of genes present in our incPRINT library. Number of interacting proteins per term and the corresponding *P*-values are indicated. (f) Biological process GO terms enriched for *Xist* region-specific interactors. Background reference population is the full set of genes present in our incPRINT library. Number of interacting proteins per term and the corresponding *P*-values are indicated.

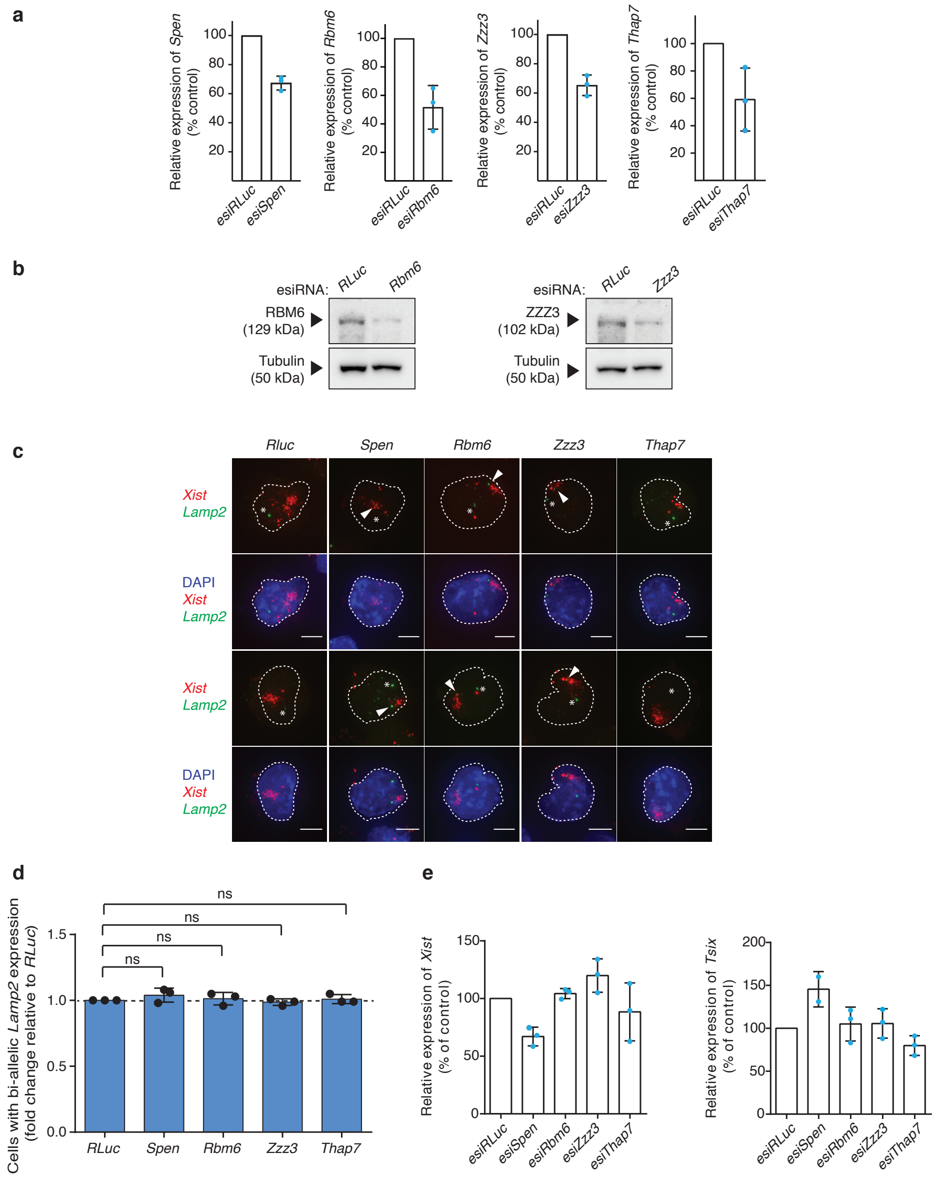

Supplementary Figure 4: Identification of proteins involved in gene silencing during XCI. (a) qRT-PCR analysis of *Spen*, *Rbm6*, and *Zzz3* levels upon their respective esiRNA-mediated depletion in TX1072 cells. *RLuc* esiRNA was used as a negative control. *β-actin* was used as a reference gene. Data from three independent experiments are represented as mean ± s.d. (b) Western blot analysis of indicated proteins. TX1072 cells were transfected with *Rbm6* and *Zzz3* esiRNAs, *RLuc* esiRNA served as a control. Tubulin was used as a loading reference. Full blots are provided as a Source Data file. (c) Representative RNA FISH images of *Xist*-induced cells upon depletion of the indicated factors. *Xist* is shown in red and the X-linked gene *Lamp2* in green. The dashed line delineates cell nuclei. Asterisks indicate *Lamp2* expression from the active X chromosome. Arrowheads indicate *Lamp2* expression from the inactive X chromosome that escapes XCI. Scale bars, 5µm. (d) Quantification of cells with bi-allelic *Lamp2* expression in absence of *Xist* (-Dox), assessed with RNA FISH and expressed as fold ratio over *RLuc* control. Data from three independent experiments are represented as mean ± s.d.; Student's t-tests: ***P*<0.01; **P*< 0.05; not significant (ns). Dashed line delineates the level of *RLuc*. (e) qRT-PCR analysis of *Xist* and *Tsix* levels in TX1072 cells upon esiRNA-mediated depletion of the respective target genes and doxycycline induction of *Xist*. *ArpPO* was used as a reference gene. Data from three independent experiments are represented as mean ± s.d.

Xist binding proteins	incPRINT			Proteomic studies			Genetic studies	
	Xist(A)	*Xist(F)*	*Xist(C)*	McHugh *et al*, 2015	Chu *et al*, 2015	Minajigi *et al*, 2015	Moindrot *et al*, 2015	Monfort *et al*, 2015
SPEN	●			●	●	●	●	●
RALY	●			●	●	●		
SRSF10	●				●			
SRSF9	●				●	●		
SRRT	●				●			
SFPQ	●				●			
YTHDC1	●				●			
RBM15B	●					●		
RBM15	●	●		●	●	●	●	
HNRNPC	●	●		●	●		●	
SRSF3	●	●			●			
SRSF7	●	●			●			
SRSF5		●			●			
HNRNPA0		●			●	●		
SLTM		●			●			
XPO5		●				●		
CLK3	●		●			●		
SAF-A			●	●	●	●		

Supplementary Table 1: Common *Xist*-binding proteins, identified by incPRINT for the three indicated *Xist* regions and by previous studies for the full-length *Xist* transcript

- Doxycycline				
	**esiRNA**	**Number of cells with biallelic *Xist***	**Number of cells with biallelic *Lamp2***	**% of cells with biallelic *Lamp2***
FISH #1	*Rluc*	81	61	86
	Spen	78	66	93
	Rbm6	64	51	89
	Zzz3	62	49	87
	Thap7	78	62	91
FISH #2	*Rluc*	99	71	82
	Spen	105	84	88
	Rbm6	99	72	80
	Zzz3	101	80	82
	Thap7	122	88	82
FISH #3	*Rluc*	74	58	84
	Spen	45	36	82
	Rbm6	72	60	88
	Zzz3	104	77	81
	Thap7	97	75	83

+ Doxycycline				
	**esiRNA**	**Number of cells with biallelic *Xist***	**Number of cells with biallelic *Lamp2***	**% of cells with biallelic *Lamp2***
FISH #1	*Rluc*	27	72	25
	Spen	59	129	44
	Rbm6	43	81	48
	Zzz3	43	80	70*
	Thap7	64	111	26
FISH #2	*Rluc*	65	83	30
	Spen	71	102	47
	Rbm6	67	104	47
	Zzz3	74	101	46
	Thap7	63	70	27
FISH #3	*Rluc*	60	60	31
	Spen	61	80	79
	Rbm6	60	100	67
	Zzz3	63	104	66
	Thap7	67	69	46

*The RNA FISH experiment was done in a separate experimental round, which also included the RLuc control. The RLuc control showed 30% of cells with bi-allelic Lamp2 expression. The ratio of defective gene silencing presented in the Table was normalized to its respective control.

Supplementary Table 2: Quantification of cells with bi-allelic *Lamp2* expression
