# Supplementary material for: In-cell identification and measurement of RNA-protein interactions
Source: Nat Commun. 2019 Nov 22;10:5317. doi: 10.1038/s41467-019-13235-w (PMC6876571; doi:10.1038/s41467-019-13235-w)
Supplement: Supplementary file 2 — Reporting Summary [file 41467_2019_13235_MOESM2_ESM.pdf]

nature research

Corresponding author(s): Alena Shkumatava

Last updated by author(s): Sep 19, 2019

Reporting Summary

Nature Research wishes to improve the reproducibility of the work that we publish. This form provides structure for consistency and transparency in reporting. For further information on Nature Research policies, see Authors & Referees and the Editorial Policy Checklist .

Statistics

For all statistical analyses, confirm that the following items are present in the figure legend, table legend, main text, or Methods section.

n/a	Confirmed	
☐	☒	The exact sample size (*n*) for each experimental group/condition, given as a discrete number and unit of measurement
☐	☒	A statement on whether measurements were taken from distinct samples or whether the same sample was measured repeatedly
☐	☒	The statistical test(s) used AND whether they are one- or two-sided<br>*Only common tests should be described solely by name; describe more complex techniques in the Methods section.*
☐	☒	A description of all covariates tested
☒	☐	A description of any assumptions or corrections, such as tests of normality and adjustment for multiple comparisons
☐	☒	A full description of the statistical parameters including central tendency (e.g. means) or other basic estimates (e.g. regression coefficient) AND variation (e.g. standard deviation) or associated estimates of uncertainty (e.g. confidence intervals)
☒	☐	For null hypothesis testing, the test statistic (e.g. *F*, *t*, *r*) with confidence intervals, effect sizes, degrees of freedom and *P* value noted<br>*Give P values as exact values whenever suitable.*
☒	☐	For Bayesian analysis, information on the choice of priors and Markov chain Monte Carlo settings
☒	☐	For hierarchical and complex designs, identification of the appropriate level for tests and full reporting of outcomes
☐	☒	Estimates of effect sizes (e.g. Cohen's *d*, Pearson's *r*), indicating how they were calculated

Our web collection on statistics for biologists contains articles on many of the points above.

Software and code

Policy information about availability of computer code

Data collection	n/a
Data analysis	n/a

For manuscripts utilizing custom algorithms or software that are central to the research but not yet described in published literature, software must be made available to editors/reviewers. We strongly encourage code deposition in a community repository (e.g. GitHub). See the Nature Research guidelines for submitting code & software for further information.

Data

Policy information about availability of data

All manuscripts must include a data availability statement. This statement should provide the following information, where applicable:

- Accession codes, unique identifiers, or web links for publicly available datasets
- A list of figures that have associated raw data
- A description of any restrictions on data availability

A data availability statement is included in the manuscript; there is no restriction on data availability.

Field-specific reporting

Please select the one below that is the best fit for your research. If you are not sure, read the appropriate sections before making your selection.

☒ Life sciences ☐ Behavioural & social sciences ☐ Ecological, evolutionary & environmental sciences

For a reference copy of the document with all sections, see nature.com/documents/nr-reporting-summary-flat.pdf

Life sciences study design

All studies must disclose on these points even when the disclosure is negative.

Sample size	The number of cells necessary for the FISH experiments was chosen based on the analyses performed Chaumeil et al, Genes and Dev 2006; approximately 100 cells were counted in each experimental group for each replicate.
Data exclusions	No data was excluded.
Replication	All attempts of replication were successful. The exact number of replicates for each experiment is indicated in either the Methods and/or in the Figure legends.
Randomization	To control for covariates, control and experimental samples were always processed in parallel.
Blinding	The FISH results were analyzed by two researchers, blind to the identify of the interrogated genes.

Reporting for specific materials, systems and methods

We require information from authors about some types of materials, experimental systems and methods used in many studies. Here, indicate whether each material, system or method listed is relevant to your study. If you are not sure if a list item applies to your research, read the appropriate section before selecting a response.

Materials & experimental systems

n/a	Involved in the study	
☐	☒	Antibodies
☐	☒	Eukaryotic cell lines
☒	☐	Palaeontology
☒	☐	Animals and other organisms
☒	☐	Human research participants
☒	☐	Clinical data

Methods

n/a	Involved in the study	
☒	☐	ChIP-seq
☒	☐	Flow cytometry
☒	☐	MRI-based neuroimaging

Antibodies

Antibodies used	anti-FLAG M2 antibody (F1804, Sigma), HRP-conjugated anti-FLAG antibody (ab1238, Abcam), anti-RBM6 (HPA026272, Sigma), anti-ZZZ3 (SAB4501106, Sigma), anti-Tubulin (CP06 mouse monoclonal DM1A, Merck Millipore).
Validation	Anti-FLAG M2 antibody is validated for Western blots and immunoprecipitation as described by the provider.<br>HRP-conjugated anti-FLAG antibody is validated for Western blot, ELISA and immunohistochemistry applications, as described on the Abcam website. Western blot experiments reveal the presence of one single specific band.<br>Anti-RBM6 (HPA026272, Sigma) is validated for Western blot and immunohistochemistry on the Sigma website. In the current study, we used this antibody for Western blot analysis. We obtained an additional lower band, but both protein size and the decreased protein levels upon knockdown allowed us to easily distinguish the correct band.<br>The same is true for the anti-ZZZ3 (SAB4501106, Sigma) antibody, except for the applications described by Sigma, which are Western blot and ELISA.

Eukaryotic cell lines

Policy information about cell lines

Cell line source(s)	TX1072 mouse ES cell line was established in the Edith Heard laboratory, Institut Curie; HEK293T cells were a gift from the Raphael Margueron lab, Institut Curie.
Authentication	All culture conditions are described in the Methods section; the TX1072 cell line was characterized in Schulz et al., 2014; Zylics et al., 2019.
Mycoplasma contamination	All cells tested negative for mycoplasma.
Commonly misidentified lines (See ICLAC register)	n/a
