# Supplementary material for: In-cell identification and measurement of RNA-protein interactions
Source: Nat Commun. 2019 Nov 22;10:5317. doi: 10.1038/s41467-019-13235-w (PMC6876571; doi:10.1038/s41467-019-13235-w)
Supplement: Supplementary file 3 — Description of Additional Supplementary Files [file 41467_2019_13235_MOESM3_ESM.docx]

**Description of Additional Supplementary Files**

File name: Supplementary Data 1
Description: incPRINT data set for *Firre.* For the status of the detected signal (columns indicated in orange), 1 = valid signal, 2 = insufficient expression levels of the test protein; 3 = invalid signal due to a high discrepancy between duplicates.

File name: Supplementary Data 2
Description: ENCODE eCLIP dataset for *Firre* and *Xist*

File name: Supplementary Data 3
Description: All Pfam protein domains used in the study

File name: Supplementary Data 4
Description: incPRINT data set for *Xist(A), Xist(F), and Xist(C).* For the status of the detected signal (columns indicated in orange), 1 = valid signal, 2 = insufficient expression levels of the test protein; 3 = invalid signal due to a high discrepancy between duplicates.

File name: Supplementary Data 5
Description: Oligonucleotide sequences
